# Intranasal Vaccination with rePcrV Protects against *Pseudomonas aeruginosa* and Generates Lung Tissue-Resident Memory T Cells

**DOI:** 10.1155/2022/1403788

**Published:** 2022-11-26

**Authors:** Yangxue Ou, Ying Wang, Ting Yu, Zhiyuan Cui, Xin Chen, Weijun Zhang, Quanming Zou, Jiang Gu, Qianfei Zuo

**Affiliations:** ^1^Department of Microbiology and Biochemical Pharmacy, College of Pharmacy, Army Medical University, 400038 Chongqing, China; ^2^953th Hospital, Shigatse Branch, Xinqiao Hospital, Army Medical University, 857000 Shigatse, China

## Abstract

Tissue-resident memory T (T_RM_) cells are immune sentinels that bear a key role in the local immune system and rapidly respond to infection. Our previous studies showed that mucosal immunization via intranasal pathways was more effective than intramuscular route. However, the mechanism of enhanced protective immunity remains unclear. Here, we formulated a *Pseudomonas aeruginosa* vaccine composed of type III secretion protein PcrV from *P. aeruginosa* and curdlan adjuvant and then administered by the intranasal route. Flow cytometry and immunofluorescence staining showed that the ratio of CD44^+^CD62L^−^CD69^+^CD4^+^ T_RM_ cells induced by this vaccine was significantly increased, and IL-17A production was notably enhanced. Further analysis revealed that vaccinated mice can protect against the *P. aeruginosa* challenge even after administration with FTY720 treatment. What is more, our results showed that CD4^+^ T_RM_ might be involved in the recruitment of neutrophils and provided partial protection against *Pseudomonas aeruginosa*. Taken together, these data demonstrated that CD4^+^ T_RM_ cells were elicited in lung tissues after immunization with rePcrV and contributed to protective immunity. Furthermore, it provided novel strategies for the development of vaccines for *P. aeruginosa* and other respiratory-targeted vaccines.

## 1. Introduction

*Pseudomonas aeruginosa* (*P. aeruginosa*), a prevalent opportunistic pathogen and Gram-negative bacteria, tends to cause acute and chronic severe pulmonary infections [1–3]. *P. aeruginosa* infections are particularly problematic in mechanically ventilated patients, chronic obstructive pulmonary disease (COPD) patients, and cystic fibrosis (CF) patients [4–9]. Recently, the emergence of multidrug-resistant (MDR) *P. aeruginosa* has become a serious clinical challenge, posing a serious threat to effective infection control in clinical [9–11]. Over the past decades, enormous efforts have been focused on *P. aeruginosa* vaccines. Regrettably, no approved vaccines are available for treatment of *P. aeruginosa* infections [12], because of its high diversity and variability.

Tissue-resident memory (T_RM_) cells are a new subpopulation of memory T cells recently identified, which embedded within peripheral tissues [13–15]. T_RM_ cells serve as immune sentinels at the respiratory tract and provide rapid and broad-spectrum protective effects against a variety of respiratory infection pathogens [15–17]. Induction of memory T and B cells has now been widely accepted as the principal disciplines for effective vaccine design which could provide robust protective immunity against pathogens caused by prior infection [18–20]. Both CD4 and CD8 T_RM_ reside in mucosal could be produced by natural infection [21]; however, natural infection could be lethal. Thus, finding an effective way to induce highly protective T_RM_ cells could be an ideal choice especially for the prevention of *P. aeruginosa*. Previous study showed the type of vaccines and adjuvants, and the route of vaccination could influence the efficacy of T_RM_. For pulmonary infectious diseases, mucosal immunization via the intranasal pathways is more effective than intramuscular route in inducing and stimulating immune protection of T_RM_ [20, 22].

Th17 has been regarded as a major player in the anti-*P. aeruginosa* immunity; indeed, in our previous study, we identified a soluble *P. aeruginosa* antigen called rePcrV which could induce Th17 response and provide protection against *P. aeruginosa* by intranasal immunization [23]. Another substrate, 1,3-*β*-glucan, derived from *Alcaligenes faecalis*, has also been reported to prompt a Th1/Th17 response [24, 25]. Therefore, we combined rePcrV and 1,3-*β*-glucan supplemented with curdlan as an adjuvant. After immunization with the vaccine by intranasal administration, we observed that the ratio of CD44^+^CD62L^−^CD69^+^CD4^+^ T_RM_ cells induced by this vaccine was significantly increased, and IL-17A production of this subpopulation was notably enhanced after in vitro stimulation. Vaccinated mice infected with *P. aeruginosa* showed a sharp reduction in the bacterial burden. What is more, our results showed that CD4^+^ T_RM_ may involve the recruitment of neutrophils and provide partial protection against *P. aeruginosa*. Better understanding the underline mechanism could provide new strategies for the development of vaccines for *P. aeruginosa* and other respiratory-targeted vaccines.

## 2. Materials and Methods

### 2.1. Animals and Strains

Adult female C57BL/6 mice (6-8 weeks) were purchased from Beijing HFK Bioscience Limited Company. Adult female CD8 KO (Cd8a^tm1Mak^) mice and adult female *μ*MT mice were obtained from Army Medical University. Adult female CB-17 SCID mice (CB17/Icr-Prkdc^scid^/IcrlcoCrl) were purchased from Beijing Vital River Laboratory Animal Technology Co., Ltd. Mice were bred in-house under specific pathogen-free (SPF) conditions at Army Medical University, Department of Microbiology and Biochemical Pharmacy. All animal studies were approved by the Animal Ethical and Experimental Committee of the Army Medical University. *P. aeruginosa* XN-1 was isolated in the Southwest Hospital of Army Medical University.

### 2.2. Immunization Procedure

For active immunization, adult female mice were vaccinated intranasally (i.n.) with 20 *μ*L of curdlan (10 mg/mL, Sigma) or purified proteins (25 *μ*g/mouse) plus curdlan (10 mg/mL, Sigma), on days 0, 14, and 21. Mice were challenged at day 35 and were anesthetized with isoflurane or pentobarbital sodium followed by the intratracheal injection of *P. aeruginosa* XN-1. The lethal dose of *P. aeruginosa* XN-1 was 1.0 × 10^7^ CFU per mouse. The sublethal dose of *P. aeruginosa* XN-1 was 1.3 × 10^6^ CFU per mouse.

### 2.3. FTY720 Treatment

FTY720 (Cayman Chemical) dissolved in saline was continuously administered i.p. (0.5 mg/kg) to mice for a period of 7 d before infection [26].

### 2.4. Isolation of Lung Lymphocyte

At day 36 after treatment, mice were sacrificed under overdose isoflurane. The lungs were dissociated with collagenase D (150 UmL^−1^, Gibco) and DNase I (1 unit/*μ*L, Sigma) at 37°C on a rocker at 260 rpm for 1 hour. Then, lung tissues were transferred to a 70 *μ*m cell strainer (Beyotime) to obtain cell suspensions. Monocytes were separated using by Percoll (Cytiva) [27].

### 2.5. Flow Cytometry

Mice were intravenously injected with 3 *μ*g APC/Cy7 anti-mouse CD45 (BioLegend) diluted in 300 *μ*L saline [28], 10 min before euthanasia. Then, lung mononuclear cells were stimulated with leukocyte activation cocktail, with BD GolgiPlug (BD Pharmingen™) for 4-6 h. PerCP/Cyanine5.5 anti-mouse CD4 (BioLegend), PE/Cy7 anti-mouse CD44 (BioLegend), FITC anti-mouse CD69 (BioLegend), and PE anti-mouse CD62L (BioLegend) were used for cell surface marker staining. APC anti-mouse IL-17A (BioLegend) and Brilliant Violet™510 anti-mouse IFN-*γ* (BioLegend) were used for intracellular staining. Zombie NIR™ Fixable Viability Kit (BioLegend) was used to distinguish between living and dead cells. For RNA-profiling, CD4^+^CD44^+^CD69^+^CD62L^−^ cells were sorted into DMEM (Gibco) with 20% fetal bovine serum (FBS, Gibco) on ice using BD FACS Aria II SORP before RNA extraction.

### 2.6. Real-Time PCR

RNA was extracted from sorted CD4^+^CD44^+^CD69^+^CD62L^−^ cells using MicroElute Total RNA Kit (OMEGA) according to the manual and stored at -80°C. *Hobit*, *Blimp-1*, *RORγt*, and *T-bet* were quantified using QuantiTect Probe RT-PCR Kit (200) (Qiagen) with SYBR-Green. The primers used were as follows: *Hobit*, forward: 5′-CTCAGCCACTTGCAGACTCA-3′, reverse: 5′-CTGTCGGTGGAGGCTTTGTA-3′; *Blimp-1*, forward: 5′-TTCTCTTGGAAAAACGTGTGGG-3′, reverse: 5′-GGAGCCGGAGCTAGACTTG-3′; *RORγt*, forward: 5′-CAGAGGAAGTGTCAGAGGCT-3′, reverse: 5′-TGCAAATGTGAAGTGCCAGC-3′; and *T-bet*, forward: 5′-CATGCCAGGGAACCGCTTAT-3′, reverse: 5′-TTGGAAGCCCCCTTGTTGTT-3′.

### 2.7. Histology and Immunofluorescence

The lungs were collected and fixed in 4% paraformaldehyde (Biosharp) and embedded in paraffin. Pathological changes were evaluated by hematoxylin and eosin stain (H&E stain) [29]. Anti-CD69 and anti-IL-17A were used for immunofluorescence staining of lung samples.

### 2.8. IL-7, IL-17A, and IFN-*γ* Neutralization

Mice were administrated 50 *μ*g/mouse of an IL-7-neutralizing antibody (BioXCell, clone M25) at days 27, 30, 32, and 34 of the first immunization (day 0) [30]. IL-17A was blocked using 300 *μ*g anti-mouse IL-17A mAb [31] (BioLegend, Clone TC11-18H10.1) administered i.v. into mice 2 d before *P. aeruginosa* XN-1 infection (at days 33 and 34). For neutralization of IFN-*γ*, mice were given intravenous injection 2 days of 300 *μ*g anti-mouse IFN-*γ*mAb [32] (BioLegend, Clone R4-6A2) before *P. aeruginosa* XN-1 infection (at days 33 and 34).

### 2.9. Neutrophil Depletion

Mice were daily injected intraperitoneally (i.p.) with anti-Ly6G antibody (BioXCell, clone 1A8, 50 *μ*g/mouse) for a period of 7 d before challenge [33] (at days 28, 29, 30, 31, 32, 33, and 34).

### 2.10. Statistical Analysis

Data are presented as mean ± SEM. Student's *t*-test and Mann–Whitney *U* test were conducted, according to the data distribution. The survival rate was analyzed by the Kaplan-Meier survival curves. GraphPad Prism 8.0 (GraphPad Software) was used for data analyses. *P* values less than 0.05 were considered significant.

## 3. Results

### 3.1. Intranasal Vaccination with rePcrV Enhanced Protection against *P. aeruginosa* Compared with Intramuscular Vaccination

**Figure 1:**
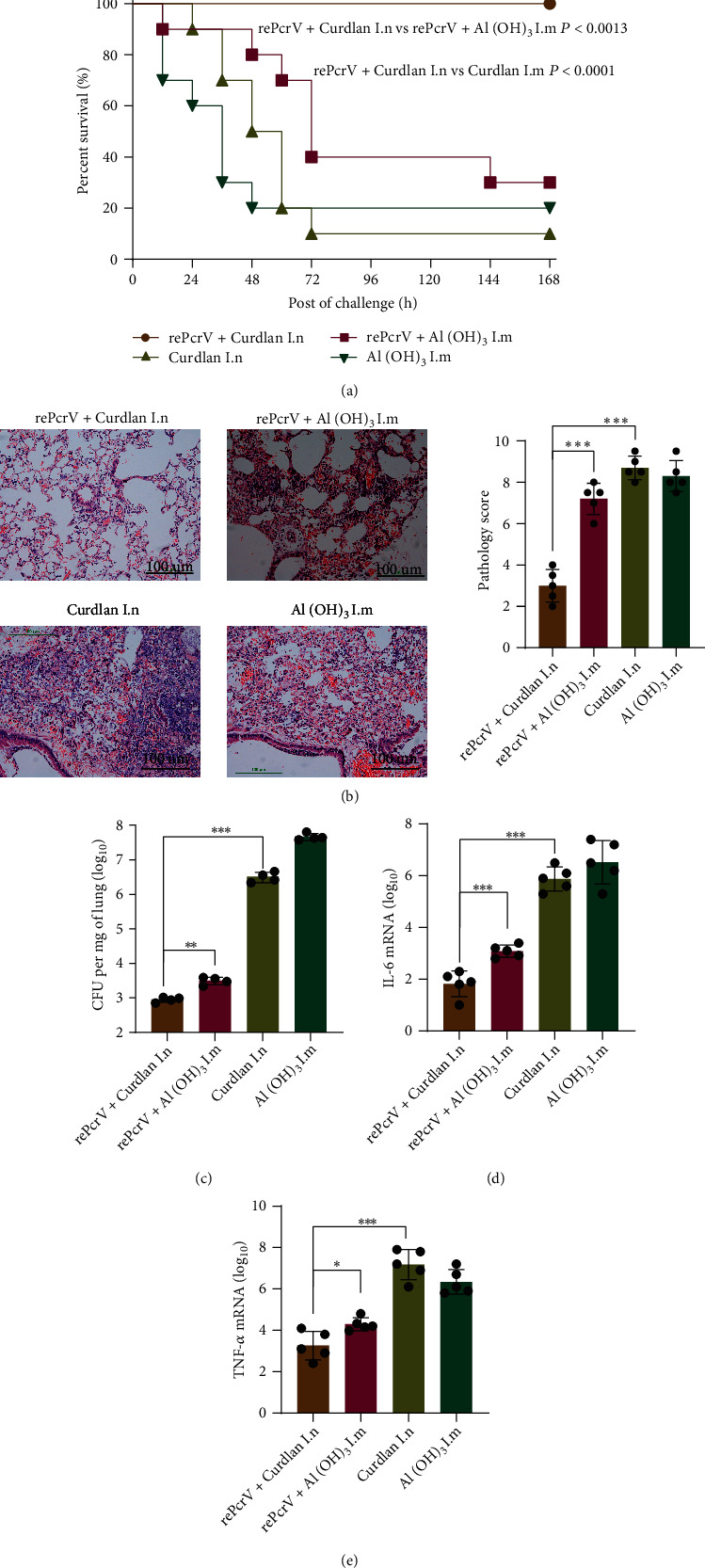
Intranasal vaccination (i.n.) with rePcrV enhanced protection compared with intramuscular vaccination (i.m.). (a) Schematic of the experimental protocol. The survival of immunized mice after challenge with the lethal dose (1.0 × 10^7^ CFU) of *P. aeruginosa* XN-1 (*n* = 10). (b) H&E (hematoxylin and eosin) stain and histology scoring of pathology in lung tissues of immunized rePcrV + curdlan mice, immunized rePcrV + Al(OH)_3_ mice, immunized curdlan mice, and vaccinated Al(OH)_3_ mice (*n* = 5). (c) Lung CFU in vaccinated rePcrV + curdlan mice, vaccinated rePcrV + Al(OH)_3_ mice, vaccinated curdlan mice, and vaccinated Al(OH)_3_ mice (*n* = 4). (d) IL-6 mRNA level in the lungs of immunized mice (*n* = 5). (e) TNF-*α* mRNA level in the lungs of immunized mice (*n* = 5). Data are shown as mean ± SEM. Significant differences were calculated with Student's *t*-test. ^∗^*P* < 0.05; ^∗∗^*P* < 0.01; ^∗∗∗^*P* < 0.001; ^∗∗∗∗^*P* < 0.0001.

PcrV has been proved to have immune protective effect by intramuscular or intraperitoneal immunization [34, 35]. In our study, we firstly compared the immune protective effects of these two different vaccination routes, intramuscular (i.m.) vaccination with the rePcrV protein formulated with aluminum adjuvant and intranasal (i.n.) immunization with curdlan. As expected, intranasal immunization route improved the efficacy of vaccine. The survival of the i.n. was higher (*P* < 0.0013) than the rate of i.m. at day 14 postinfection (Figure 1(a)). Then, mice were administrated a sublethal dose of *P. aeruginosa*. A histological analysis of lung tissues of rePcrV i.m. suggested a further increase in inflammatory cell infiltration. Meanwhile, the rePcrV i.n. showed significant reduction (*P* < 0.001) in lung pathology score (Figure 1(b)). Furthermore, the bacterial burdens of the rePcrV i.n. were significantly decreased (rePcrV i.n. vs. rePcrV i.m. *P* < 0.01, Figure 1(c)). The mRNA expression of IL-6 (rePcrV i.n. vs. rePcrV i.m. *P* < 0.001, Figure 1(d)) and TNF-*α* (rePcrV i.n. vs. rePcrV i.m. *P* < 0.05, Figure 1(e)) was also reduced in rePcrV i.n. Thus, intranasal vaccination with rePcrV enhanced protection against *P. aeruginosa* compared with intramuscular vaccination.

### 3.2. CD4 T Cells Were Essential for rePcrV-Mediated Protection in *P. aeruginosa* Pneumonia

**Figure 2:**
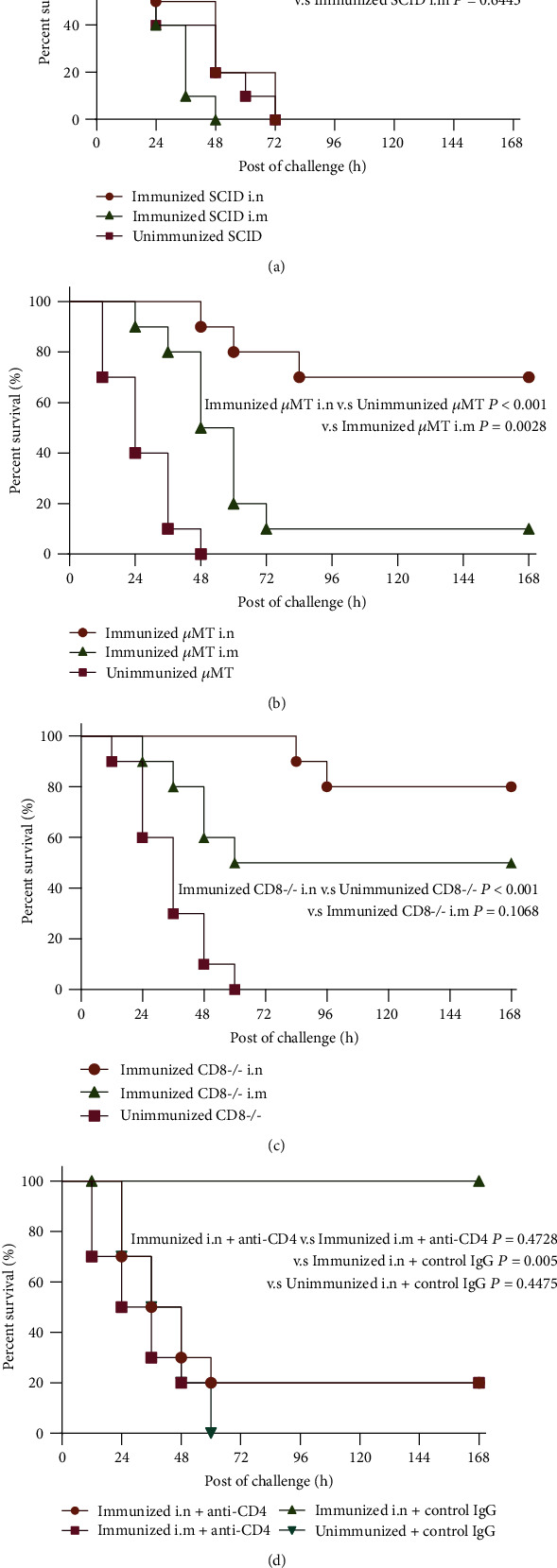
CD4 T cells are essential for rePcrV-mediated protection in *Pseudomonas aeruginosa* pulmonary infection. (a) Survival in the rePcrV + curdlan vaccinated mice (immunized SCID i.n.), rePcrV + Al(OH)_3_ mice (immunized SCID i.m.), and unimmunized SCID mice (*n* = 10). (b) Survival in the rePcrV + curdlan vaccinated mice (immunized *μ*MT i.n.), rePcrV + Al(OH)_3_ mice (immunized *μ*MT i.m.), and unimmunized *μ*MT mice (*n* = 10). (c) Survival in the rePcrV + curdlan vaccinated mice (immunized CD8^−^/^−^ i.n.), rePcrV + Al(OH)_3_ mice (immunized CD8^−^/^−^ i.m.), and unimmunized CD8^−^/^−^ mice (*n* = 10). (d) Survival in the rePcrV + curdlan vaccinated mice (immunized i.n. + anti-CD4), rePcrV + Al(OH)_3_ mice (immunized i.m. + anti-CD4), rePcrV + curdlan vaccinated mice (immunized i.n. + control IgG), and unimmunized + control IgG mice (*n* = 10). Immunized 6-8-week-old female C57 mice were intraperitoneally (i.p.) treated with 200 *μ*g of anti-GK1.5 Ab (BioXCell, to deplete CD4^+^ T cells) or isotype control (Rat IgG2b, *κ*, BioXCell) 2 days before vaccination and were administered weekly throughout the whole experiment to maintain CD4^+^ T cell depletion. All mice were challenged with 1.0 × 10^7^ CFU of the *P. aeruginosa* XN-1 strain i.n. (in the nose) and i.m. (in the muscle). ^∗∗∗^*P* < 0.001 by log-rank test.

To inquire the role of lymphocyte-mediated immune responses during rePcrV-induced protection, adult female CB-17 SCID mice were vaccinated with rePcrV plus curdlan or rePcrV plus aluminum. Mice were challenged with *P. aeruginosa* XN-1 and were observed to survive for 14 days. As shown in Figure 2(a), there was no statistical difference (*P* = 0.1316) in survival rate between rePcrV-immunized SCID mice and -unimmunized mice, indicating that a complete lymphocyte system was required for protection after rePcrV immunization in *P. aeruginosa* pneumonia. In order to determine the relative requirements for humoral immunity and cellular immunity, rePcrV vaccine tested the protection in *μ*MT mice (which lack mature B cells), CD8 T cell KO mice, and CD4-depleted mice (by intraperitoneal injection of anti-CD4 antibody GK1.5). As shown in Figure 2(b), rePcrV-immunized *μ*MT mice were significantly protected (*P* < 0.001) after *P. aeruginosa* XN-1 challenge, compared with unimmunized mice which were not protected. The result of CD8 T cell KO mice was the same (*P* < 0.001, Figure 2(c)). However, the rePcrV-immunized CD4-depleted mice (*P* = 0.4728, Figure 2(d)) were not protected after *P. aeruginosa* XN-1 challenge. These data suggested the key role for CD4 T cells in mediating protection after immunization with rePcrV.

### 3.3. Intranasal Vaccination with rePcrV Initiates the CD4^+^ T_RM_ Cell Response

**Figure 3:**
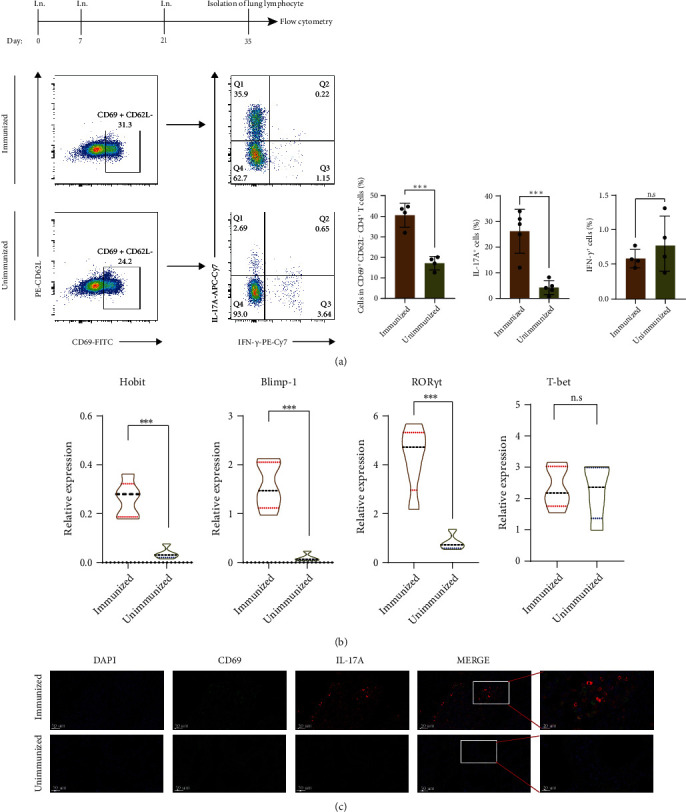
Intranasal vaccination with rePcrV primes the CD4^+^ T_RM_ cells response. (a) Schematic of the experimental protocol. Representative intracellular staining profiles and pooled data of IL-17A and IFN-*γ* in CD4^+^CD44^+^CD69^+^CD62L^−^ T cells in the lungs of immunized mice (rePcrV + curdlan i.n.) or unimmunized mice (*n* = 4). (b) *Hobit*, *Blimp-1*, *ROR-γt*, and *T-bet* expressions of CD4^+^ T cells in the lung tissues of immunized mice (rePcrV + curdlan i.n.) or unimmunized mice (*n* = 4). (c) Representative immunofluorescence images of the lung tissues stained with DAPI (blue), anti-CD69 (green), and anti-IL-17A (red) from immunized mice (rePcrV + curdlan i.n.) or unimmunized mice (*n* = 4). Data are presented as mean ± SEM. *P* values were calculated by Student's *t*-test. ^∗^*P* < 0.05; ^∗∗^*P* < 0.01; ^∗∗∗^*P* < 0.001; ^∗∗∗∗^*P* < 0.0001.

The result above showed that CD4^+^T cells are essential for the anti-*P. aeruginosa* immunity. However, it is still unknown whether circulating or resident CD4^+^ T cell is the major player. To this end, the lungs were dissociated into a single cell suspension and detected by flow cytometry. A dramatic increase in CD4^+^CD44^+^CD62L^−^CD69^+^T_RM_ cells was observed in vaccinated mice compared with unimmunized mice (*P* < 0.001, Figure 3(a)). Transcriptional analysis of T_RM_ cells showed that they expressed a unique transcription factor profile. Since *Hobit* together with *Blimp-1* regulates the differentiation and maintenance of T_RM_ cells [36], we purified T_RM_ cells from immunized or unimmunized mice and determined the level of *Hobit*, *Blimp-1*, *RORγt*, and *T-bet* mRNA. As shown in Figure 3(b), the level of *Hobit*, *Blimp-1*, and *RORγt* was increased in mice immunized with rePcrV compared with unimmunized (*P* < 0.001, respectively). To examine the expression of IL-17A production in CD4^+^ T_RM_ cells, we employed immunofluorescence staining. The result revealed that the IL-17A expression was enhanced in immunized mice (Figures 3(a) and 3(c)). Representative gating strategies were shown in figure S1.

### 3.4. CD4^+^ T_RM_ Cells Partially Protected against *Pseudomonas aeruginosa* Pulmonary Infection

**Figure 4:**
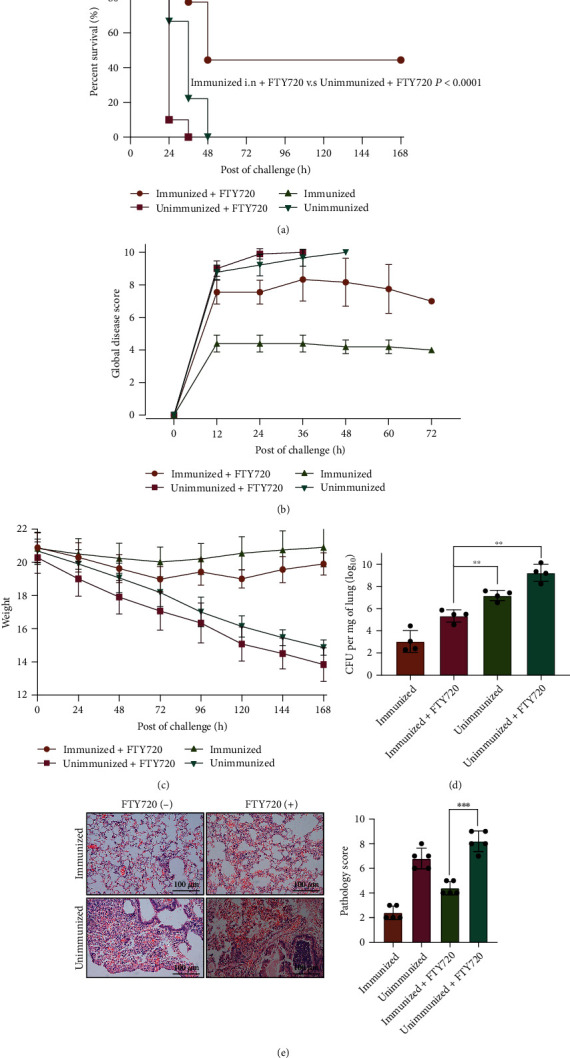
CD4^+^ T_RM_ cells protect against *Pseudomonas aeruginosa* pulmonary infection. (a) The survival of naïve C57BL/6 mice with or without FTY720 treatment and rePcrV-immunized mice with or without FTY720 treatment (*n* = 10). ^∗∗∗^*P* < 0.01 by log-rank test. (b) Global disease score in 4 groups of mice after challenge with 1.3 × 10^6^ CFU of *P. aeruginosa* XN-1 (*n* = 10). (c) Weight loss in the naïve mice with or without FTY720 treatment and rePcrV-immunized mice with or without FTY720 treatment after challenge with 1.3 × 10^6^ CFU of *P. aeruginosa* XN-1 (*n* = 10). (d) Lung CFU 24 hours after infection in the naïve mice with or without FTY720 treatment and rePcrV-immunized mice with or without FTY720 treatment (*n* = 4). (e) Representative H and E stains in naïve mice with or without FTY720 treatment and rePcrV-immunized mice with or without FTY720 treatment lung 24 hours post-*P. aeruginosa* XN-1 challenge are shown (scale bar = 100 *μ*m) (*n* = 5). Significant differences are designated by using Student's *t*-test. ^∗^*P* < 0.05; ^∗∗^*P* < 0.01; ^∗∗∗^*P* < 0.001; ^∗∗∗∗^*P* < 0.0001.

To exclude the contribution of circulating memory cells to the recall responses, we administered FTY720 [37, 38] (a S1P inhibitor that blocks the egress of T cells from repositioning from secondary lymphoid organs to the tissue). We found that FTY720 treatment followed by a *P. aeruginosa* XN-1 challenge induced higher survival in immunized mice (*P* < 0.0001) but not in unimmunized mice (Figure 4(a)). Vaccine efficacy was maintained in vaccinated mice with FTY720 treatment, as measured indirectly by global disease score (Figure 4(b)) and weight loss (Figure 4(c)). Furthermore, the bacterial load of immunized mice treated with FTY720 decreased significantly (*P* < 0.01, Figure 4(d)). In contrast, immunized mice significantly alleviated pathological damage (*P* < 0.001, Figure 4(e)). It should be noted that, compared with immunized mice without FTY720 treatment, immunized mice with FTY720 treatment diminished partial protection, which suggested that circulating T cells also played a role in preventing *P. aeruginosa* infection.

### 3.5. rePcrV Vaccine Efficacy Depended on IL-17A Expression by CD4^+^ T_RM_ Cells and Remained Independent of IL-7

**Figure 5:**
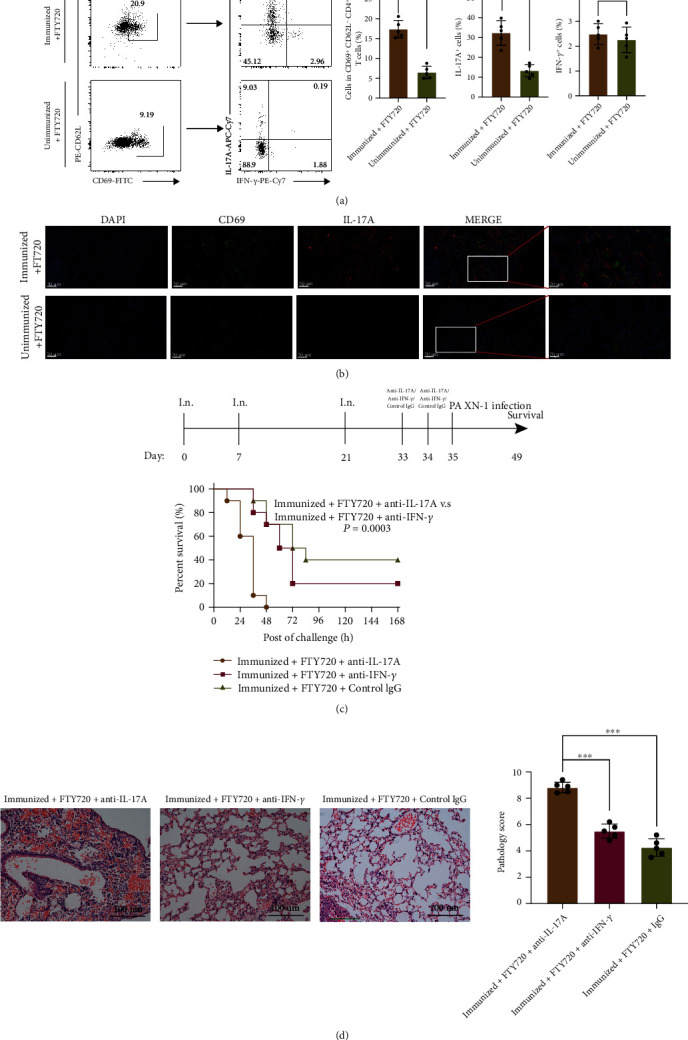
rePcrV vaccine efficacy depends on the IL-17A expression by CD4^+^ T_RM_ cells. (a) The representative dot plots showed CD4^+^CD44^+^CD69^+^CD62L^−^ T cells in the lungs of rePcrV-immunized mice with FTY720 treatment or naïve C57BL/6 mice with FTY720 treatment. The graph indicates the number of CD4^+^CD44^+^CD69^+^ CD62L^−^ T cells per mouse (*n* = 5) found in lung tissues of naive and immunized mice with FTY720 treatment. Significant differences were calculated with Mann–Whitney *U* test. (b) Representative immunofluorescence images of the lung stained with DAPI (blue), anti-CD69 (green), and anti-IL-17A (red) from naive and immunized mice with FTY720 treatment (*n* = 4). (c) Schematic of the experimental protocol. Survival in the FTY720 treatment-immunized mice with anti-IL-17A or IFN-*γ* Ab treatment (*n* = 10). ^∗∗∗^*P* < 0.001 by log-rank test. (d) Representative H and E stains in the FTY720 treatment-immunized mice with anti-IL-17A or IFN-*γ* Ab treatment (*n* = 5). Significant differences are designated by using Student's *t*-test. ^∗^*P* < 0.05; ^∗∗^*P* < 0.01; ^∗∗∗^*P* < 0.001; ^∗∗∗∗^*P* < 0.0001.

Lung CD4^+^ T_RM_ cells in vaccinated mice with FTY720 treatment showed higher level of IL-17A secretion compared with cells from FTY720-treated unimmunized mice (*P* < 0.01, Figures 5(a) and 5(b)). We treated mice with anti-IL-17A antibody and anti-IFN-*γ* antibody before and during vaccination to determine whether IL-17A or IFN-*γ* was required for rePcrV vaccine efficacy in the lungs. Anti-IL-17A-immunized mice were not protected against the challenge of *P. aeruginosa* XN-1 (Figure 5(c)). In line with this, the histological analysis of the lung tissues of anti-IL-17A-immunized mice revealed a further increase of peribronchial inflammatory cell infiltration (*P* < 0.001, Figure 5(d)).

**Figure 6:**
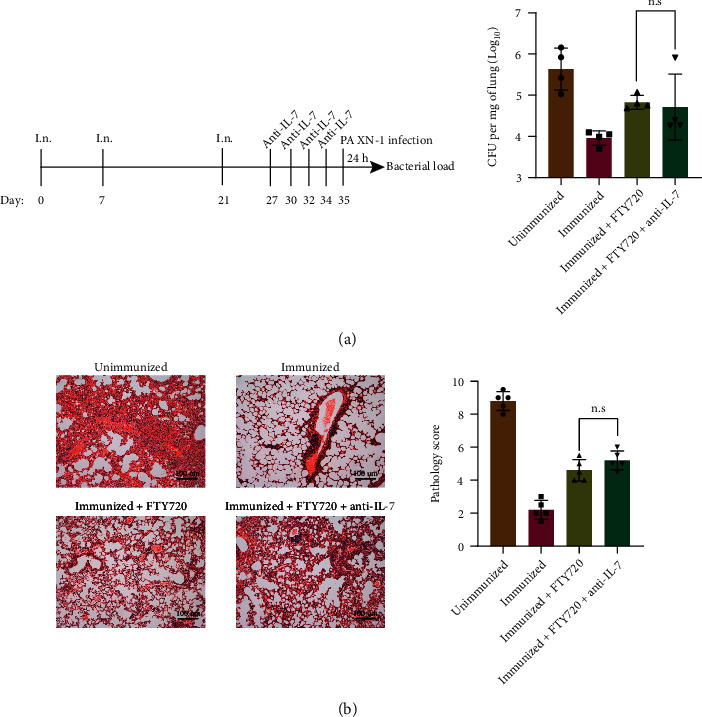
rePcrV vaccine efficacy remains independent of IL-7. (a) Experimental timeline. Lung CFU 24 hours after infection in naïve mice, rePcrV-immunized mice, and FTY720 treatment-immunized mice with or without anti-IL-7 Ab (*n* = 4). (b) Representative H and E stains in naïve mice, rePcrV-immunized mice, and FTY720 treatment-immunized mice with or without anti-IL-7 Ab (*n* = 5). Significant differences were calculated using unpaired *t*-test. The “n.s.” means “no significant difference.”

IL-7 signaling is regarded as a key mediator for homeostatic proliferation of CD4 T cells, which could explain the long-term and circulatory independent maintenance of T_RM_ cells. To assess whether IL-7 mediated the population expansion of T_RM_ cells and contributed to its survival, we applied a neutralizing antibody to IL-7 (anti-IL-7) at days 27, 30, 32, and 34 of the first immunization (day 0). The results showed that neutralization of IL-7 did not increase the bacterial load (*P* = 0.7836, Figure 6(a)), and there was no statistical difference in histopathological examination between groups (*P* = 0.1599, Figure 6(b)).

### 3.6. Depletion of Neutrophils Impaired the Clearance of *Pseudomonas aeruginosa* from the Lung

**Figure 7:**
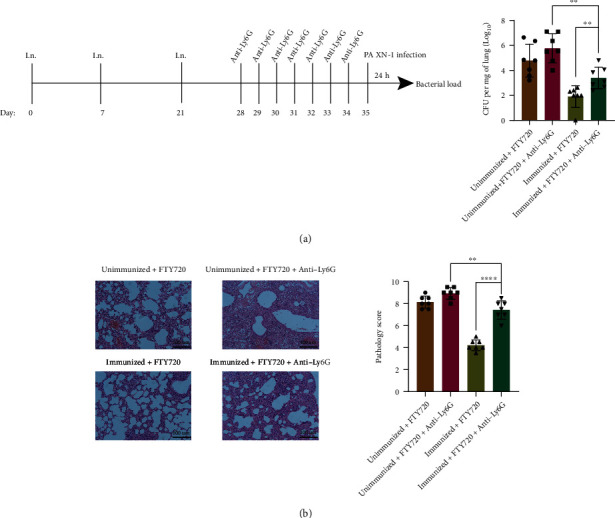
Depletion of neutrophils impairs clearance of Pseudomonas aeruginosa from the lung. (a) Experimental timeline. Lung CFU 24 hours after infection in FTY720 treatment mice with or without anti-Ly6G Ab and FTY720 treatment-immunized mice with or without anti-Ly6G Ab (*n* = 7). (b) Representative H and E stains in FTY720 treatment mice with or without anti-Ly6G Ab and FTY720 treatment-immunized mice with or without anti-Ly6G Ab (*n* = 7). Significant differences were calculated by using Student's *t*-test. ^∗^*P* < 0.05; ^∗∗^*P* < 0.01; ^∗∗∗^*P* < 0.001; ^∗∗∗∗^*P* < 0.0001.

Neutrophils are main orchestrators of lung inflammation and play a unique role in the connection between innate and adaptive immunity [39]. In order to investigate whether neutrophils play a role in CD4^+^ T_RM_ cells mediated protection against *P. aeruginosa*, neutrophils were deleted before challenge. The results showed that in the neutrophil depletion mice, CFU counts were increased in the lungs of mice treated with anti-Ly6G (*P* < 0.01, Figure 7(a)), and lung damage was worse (*P* < 0.0001, Figure 7(b)). Flow cytometry showed that neutrophil depletion did not impact the CD4^+^ T_RM_ cell population (*P* = 0.7296, Figure S2). These data indicated that CD4^+^ T_RM_ may be involved in recruitment of neutrophils and provided partial protection against *P. aeruginosa*.

## 4. Discussion

According to the different types of cytokines secreted, CD4^+^ T_RM_ cells are divided into Th1, Th2, or Th17 subtypes. Generally, CD4^+^ T_RM_ cells in viral infection and tumors mainly secreted IFN-*γ*, while CD4^+^ T_RM_ cells induced by bacterial or fungal infection mainly expressed IL-17A. The study indicated that dermal *Candida albicans* infection preferentially produces CD4^+^ IL-17A^+^ T_RM_ cells. When reinfected with *Candida albicans*, T_RM_ cells could rapidly clear infection challenges [40]. Previous work showed that lung T_RM_ cells were elicited by heat-killed *K. pneumoniae* [41]. By using IL-17A tracking-fate mouse models [42], CD4^+^ T_RM_ cells were found derived from effector Th17 cells [27]. Our previous study found that rePcrV could induce Th17 response and enhanced protection [23]. The results of this study initially demonstrate that rePcrV intranasal immunization could induce the generation of CD4^+^ T_RM_ cells secreting IL-17A in lung tissues of mice, and these cells produced a protective immune response after *P. aeruginosa* infection. Therefore, the origin of CD4^+^ IL-17A^+^ T_RM_ cells and their relationship with Th17 cells need to be further investigated in subsequent experiments.

FTY720 not only blocks the egress of T cells but also prevents migration of B cells from lymph nodes to the circulation [15, 43]. Indeed, FTY720 treatment appeared to affect bacterial burdens and survival in the immunized group, suggesting that circulating T cells or antibody-producing cells were also required in preventing *P. aeruginosa* infection. However, treatment with FTY720 did not affect T_RM_ cell expansion in the lungs. Our data showed that there was no statistical significance between mice with FTY720 treatment and mice without FTY720 treatment (Figure S3).

Long-term survival in peripheral tissues is another important characteristic of T_RM_ cells [13, 14, 44]. Furthermore, researches showed that the survival and expansion of T_RM_ cells in peripheral tissues were mainly regulated by the local immune microenvironment. The formation of the local microenvironment was associated with the involvement of multiple cytokines, such as IL-2, IL-7, IL-15, and TGF-*β* [45–47]. It also showed that multiple correlated signaling pathways may be involved in the maintenance induction of T_RM_ cells in peripheral tissues, including PI3K/Akt, JAK/STAT5, and Notch signal pathways [48]. In our research, we found that neutralization of IL-7 did not affect rePcrV vaccine efficacy, and there was no significance in the bacterial load (Figure 6). Regrettably, our work did not yet clarify the mechanisms of T_RM_ cell survival and amplification. We will continue to explore them in the future.

Studies have reported that the *acellular pertussis* vaccine vaccinated with intramuscular injection has a relatively short immunoprotection period and has no obvious effect on the colonization and transmission of *B. pertussis* in the nasal cavity [49]. On the contrary, nasal inoculation of attenuated pertussis vaccine BPZE1 can resist the infection caused *B. pertussis* [50]. Comparing the intranasal or injected influenza vaccines, we found that the route of administration and the type of vaccines (inactivated vaccine and live vaccine) also affect the production of CD4^+^ T_RM_ cells. Nasal vaccination with a live attenuated influenza vaccine (FluMist) induced antigen-specific CD4^+^ T_RM_ cells in the lung, mediating long-term protection against heterologous influenza virus strains. However, inactivated influenza virus vaccine (Fluzone) did not elicit T_RM_ cell production after nasal inoculation but induced strain-specific neutralizing antibody production [50]. Hence, choosing the appropriate vaccination route and vaccine type is an important means to induce respiratory T_RM_ cell production.

## Data Availability

All the data are included in this paper.

## Supplementary Materials

Supplementary Materials Figure S1: representative gating strategies for CD44^+^CD62L^−^CD69^+^CD4^+^ T_RM_ cells in the lungs. CD44^+^CD62L^−^CD69^+^CD4^+^ T_RM_ cells were gated on live CD45^−^ cells. Figure S2: the graph indicates the number of CD4^+^CD44^+^CD69^+^ CD62L^−^ T cells per mouse (*n* = 4) found in the lungs of FTY720 treatment-immunized mice with or without anti-Ly6G Ab. Figure S3: CD4^+^ T_RM_ cells protect against Pseudomonas aeruginosa pulmonary infection. (A) The representative dot plots showed CD4^+^CD44^+^CD69^+^CD62L^−^ T cells in the lungs of rePcrV-immunized mice with or without FTY720 treatment. (B) The graph indicates the number of CD4^+^CD44^+^CD69^+^ CD62L^−^ T cells per mouse (*n* = 4) found in the lungs of immunized mice with or without FTY720 treatment.
