# Supplementary material for: Intranasal Vaccination with rePcrV Protects against Pseudomonas aeruginosa and Generates Lung Tissue-Resident Memory T Cells
Source: J Immunol Res. 2022 Nov 26;2022:1403788. doi: 10.1155/2022/1403788 (PMC9719435; doi:10.1155/2022/1403788)
Supplement: Supplementary Materials — Figure S1: representative gating strategies for CD44+CD62L−CD69+CD4+ TRM cells in the lungs. CD44+CD62L−CD69+CD4+ TRM cells were gated on live CD45− cells. Figure S2: the graph indicates the number of CD4+CD44+CD69+ CD62L− T cells per mouse (n = 4) found in the lungs of FTY720 treatment-immunized mice with or without anti-Ly6G Ab. Figure S3: CD4+ TRM cells protect against Pseudomonas aeruginosa pulmonary infection. (A) The representative dot plots showed CD4+CD44+CD69+CD62L− T cells in the lungs of rePcrV-immunized mice with or without FTY720 treatment. (B) The graph indicates the number of CD4+CD44+CD69+ CD62L− T cells per mouse (n = 4) found in the lungs of immunized mice with or without FTY720 treatment. [file 1403788.f1.docx]

**Supplementary Materials for**

**Intranasal vaccination with rePcrV protects against *Pseudomonas aeruginosa* and generate lung tissue-resident memory T cells**


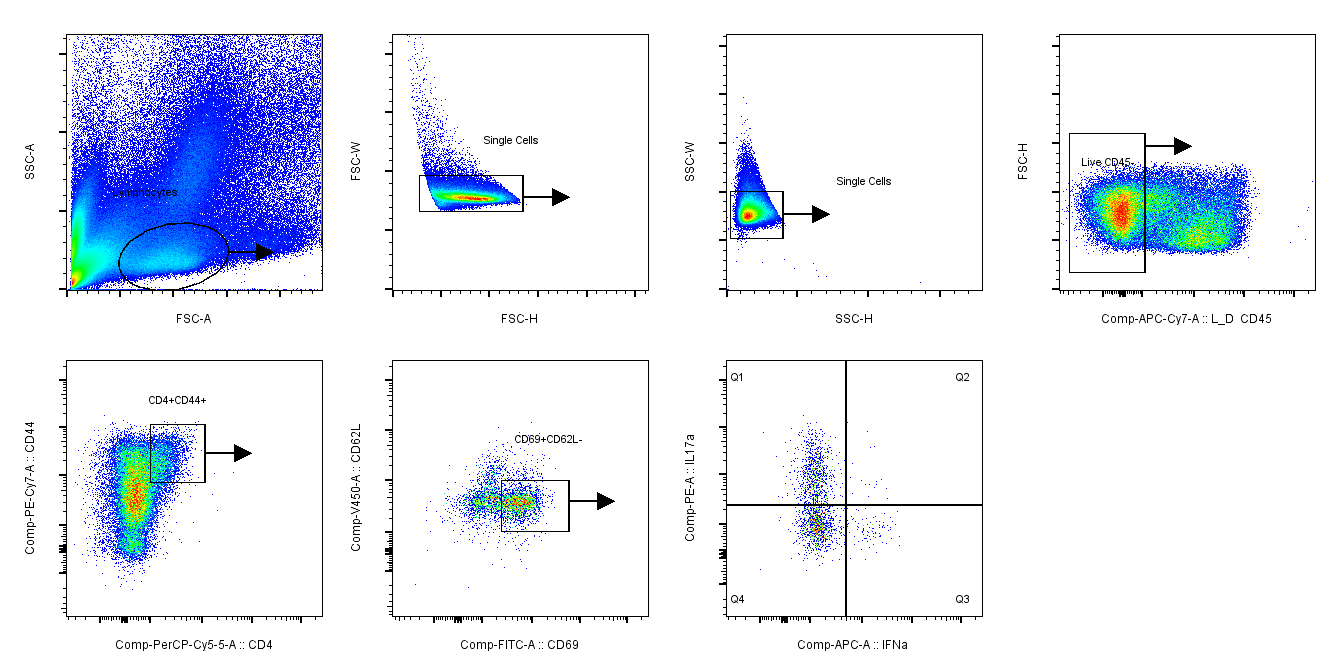


Fig.S1 Representative gating strategies for CD44^+^CD62L^-^CD69^+^CD4^+^ T_RM_ cells in lungs. CD44^+^CD62L^-^CD69^+^CD4^+^ T_RM_ cells were gated on live CD45^-^ cells.


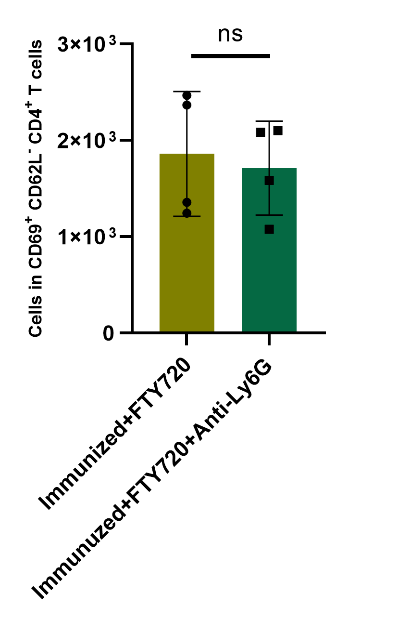


Fig.S2 The graph indicates the number of CD4^+^CD44^+^CD69^+^ CD62L^-^ T cells per mouse (n = 4) found in lungs of FTY720 treatment immunized mice with or without anti-Ly6G Ab.

**A B**


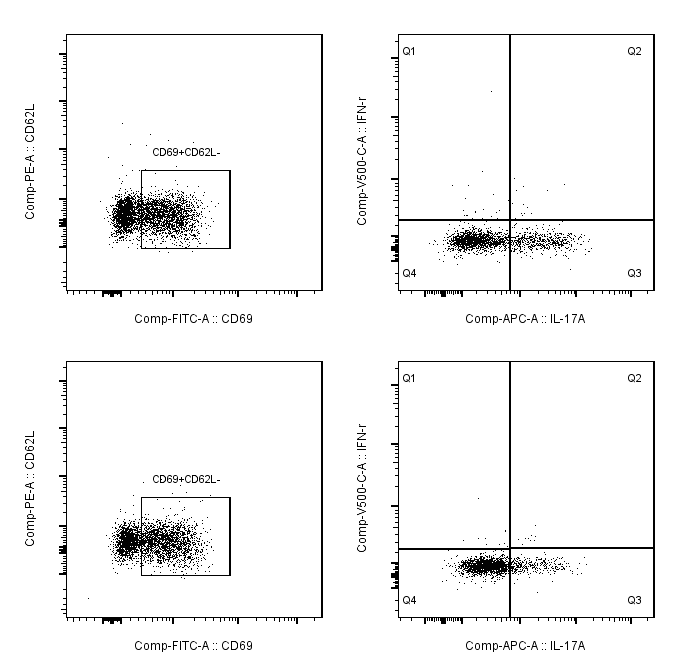

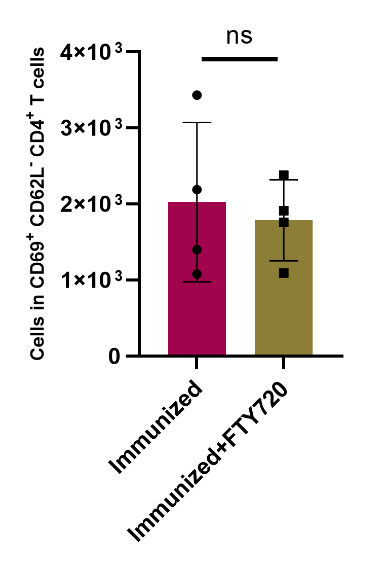


Fig.S3| CD4^+^ TRM cells protect against Pseudomonas aeruginosa Pulmonary infection. (A) The representative dot plots showed that CD4^+^CD44^+^CD69^+^CD62L^-^ T cells in the lungs of rePcrV immunized mice with or without FTY720 treatment. (B) The graph indicates the number of CD4^+^CD44^+^CD69^+^ CD62L^-^ T cells per mouse (n = 4) found in lungs of immunized mice with or without FTY720 treatment.
